# Conetworks and Organogels of Poly(methyl acrylate) Crosslinked with Degradable, Disulfide and β-Thioester Containing Bisacrylate Macromonomer

**DOI:** 10.3390/gels12100862

**Published:** 2026-09-24

**Authors:** Aiman Aitkazina, Ákos Szabó, László Trif, Klára Verebélyi, Dóra Fecske, Anna Petróczy, Béla Iván

**Affiliations:** 1 Polymer Chemistry and Physics Research Group, Institute of Materials and Environmental Chemistry, Research Centre for Natural Sciences, Magyar tudósok körútja 2, H-1117 Budapest, Hungary; 2 George Hevesy PhD School of Chemistry, Institute of Chemistry, Faculty of Science, Eötvös Loránd University, Pázmány Péter sétány 1/A, H-1117 Budapest, Hungary; 3 Functional Nanoparticles Research Group, Institute of Materials and Environmental Chemistry, Research Centre for Natural Sciences, Magyar tudósok körútja 2, H-1117 Budapest, Hungary

**Keywords:** polymer conetwork, organogel, disulfide, β-thioester by thiol–ene Michael addition, acrylate-telechelic sulfur-rich macrocrosslinker, poly(methyl acrylate), chemically forced blending, high thermal stability, selective swelling, degradability

## Abstract

Here, we report, for the first time, on a new class of sulfur-containing double hydrophopbic conetworks, poly(methyl acrylate)-*l*-poly(3,6-dioxa-1,8-octane-dithiol-*co*-poly(ethylene glycol) diacrylate) (PMA-*l*-P(DODT-*co*-PEGDA). Direct one-pot synthesis of acrylate-telechelic P(DODT-*co*-PEGDA) macromonomer in a gram-scale for the preparation of the conetworks was obtained by copolymerization of DODT with PEGDA via simultaneous oxidative disulfide formation and thiol–ene Michael addition click reactions. Conetworks with a broad composition range of 20–70 wt% sulfur-rich P(DODT-*co*-PEGDA) macrocrosslinker contents were obtained by radical copolymerization. High gel fractions and composition near to that of the feed ratios verified successful conetwork synthesis. DSC analysis indicates forced blending of the immiscible PMA and P(DODT-*co*-PEGDA) in the conetworks by one single *T*_g_, which deviates from both the additive rule and the Fox equation for miscible blends. These novel conetworks exhibit high thermal stability up to ~300 °C and independent decomposition patterns of the components in two separate steps. The PMA-*l*-P(DODT-*co*-PEGDA) conetworks behave as organogels by swelling in organic solvents, such as THF, acetone, toluene, with composition dependent swelling degrees up to ~900%. Degradation by breaking the disulfide bonds with thiols followed by hydrolysis with NaOH led to complete disintegration of the PMA-*l*-P(DODT-*co*-PEGDA) conetworks. These findings enable establishing a new platform for the rational design of a variety of novel sulfur-containing functional and degradable macromolecular assemblies.

## 1. Introduction

Polymer conetworks and their gels have attracted significant interest in recent years (see e.g., Refs. [1,2,3,4,5,6,7,8,9,10,11,12,13,14,15,16,17,18,19,20,21,22,23,24,25,26,27,28,29,30,31,32,33] and references therein). These macromolecular assemblies are composed of at least two different polymer chains interconnected with covalent or supramolecular bonds in a single network structure. Among such materials, intensive research has been focused on amphiphilic conetworks [1,2,3,4,5,6,7,8,9,10,11,12,13,14,15,16,17,18,19,20,21,22,23,24], consisting of immiscible hydrophilic and hydrophobic polymer chains, due to their unique nanophase separated morphologies [23,24], properties and advanced application possibilities, ranging from organic solar cells [5] to polymer electrolytes in Li-ion batteries [10], various biomaterials [6,7,9,11], antibiofouling [8,13,14,16] and antimicrobial [22] surfaces, conetwork materials and gels with enhanced physical properties [9,17,20], responsive gels [11,18], amphiphilic microcapsules [19], etc., while much less effort has been devoted to exploring double-hydrophobic co-networks with immiscible components [25,26,27,28] on the one hand. On the other hand, although recent advances in sulfur-containing polymers and networks, especially with degradable disulfide or β-thioester linkages, have led to a large variety of macromolecules and polymer networks by various polymerization techniques, such as thiol–ene reactions by radical or Michael addition processes, polymerization of dithiolanes, chain and step growth polymerizations of functional monomers with sulfur content, etc. (see e.g., Refs. [34,35,36,37,38,39,40,41,42,43,44,45,46,47,48,49,50,51,52,53,54,55,56] and references therein), only sporadic reports can be found in the literature on conetworks with structural units with sulfur-containing moieties [30,31,32,33]. Kong et al. used a disulfide-containing initiator to prepare azide-telechelic poly(ε-caprolactone) (PCL), which was used to crosslink alkyne-ended tetrafunctional poly(ethylene glycol) (PEG) by an azide-alkyne click reaction to obtain amphiphilic conetworks with a disulfide linkage in the PCL chains [30]. Treatment of these conetworks with excess dithiol (dithiothreitol, DTT) led to scission by the reduction of the disulfide units in the conetwork. Wu and coworkers reported on hybrid conetworks between a polydisulfide of thioctic acid and Fe^3+^ ions via complexation [31]. Kyu et al. prepared a conetwork of thiol-ended triarm star polydisulfide with acrylate-terminated triarm star PEG by radical thiol–ene reaction, and investigated this amphiphilic conetwork as polyelectrolyte in Li-ion batteries [32,33].

In this study, we report on the synthesis of novel sulfur-containing conetworks and their organogels composed of poly(methyl acrylate) (PMA) crosslinked with acrylate-telechelic poly(3,6-dioxa-1,8-octane-dithiol-*co*-poly(ethylene glycol) diacrylate) (P(DODT-*co*-PEGDA)) copolymer via radical copolymerization, that is, by the so called macromonomer method of conetwork synthesis. The P(DODT-*co*-PEGDA) copolymer was obtained by simultaneous oxidative disulfide formation and thiol–ene Michael addition reaction by reacting DODT with PEGDA, leading to a macromolecule with both disulfide and β-thioester linkages and acrylate termini [55]. The gel fractions, composition, swelling behavior as organogels and thermal characteristics of the resulting double hydrophobic PMA-*l*-P(DODT-*co*-PEGDA) conetworks were determined (-*l*- stands for “*linked by*”). Due to the presence of the degradable disulfide and β-thioester units in the macrocrosslinker in these conetworks, degradation by reduction and hydrolysis, respectively, is demonstrated to break the crosslinked structure. These novel sulfur-containing conetworks with targeted composition, swelling properties and degradation behavior enable a broad range of application possibilities, such as additives or membranes in Li-sulfur batteries, coatings with dynamic disulfide linkages, drug release matrices, heavy metal extraction materials for environmental protection purposes, nanohybrids with inorganic nanoparticles, etc., and these materials may also offer the basis for various other sulfur-containing macromolecular assemblies and gels which have not existed before.

## 2. Results and Discussion

### 2.1. Synthesis of the PMA-l-P(DODT-co-PEGDA) Conetworks

Scheme 1 shows the synthetic route and conditions for the preparation of PMA-*l*-P(DODT-*co*-PEGDA) conetworks. This was preceded by the synthesis of the acrylate-telechelic P(DODT-*co*-PEGDA) macromonomer. As preliminary results in our laboratories indicated, reacting a stoichiometric amount of DODT and PEGDA in the presence of H_2_O_2_ as oxidant and PMDETA as a base catalyst in THF at room temperature leads to the acrylate-ended P(DODT-*co*-PEGDA) macrocrosslinker with disulfide and β-thioester linkages in the chain by simultaneous oxidative disulfide formation via thiol–thiol coupling of DODT and thiol–ene Michael addition reaction between DODT and the acrylate groups of PEGDA [55]. Scaling up this process to gram-scale for obtaining sufficient amount of macrocrosslinker proved to also be successful, as GPC and ^1^H NMR analyses of the resulting copolymer indicate. As shown by the GPC chromatogram in Figure 1, a P(DODT-*co*-PEGDA) copolymer with monomodal and relatively narrow molecular weight distribution is formed. The evaluation of the chromatogram resulted in *Đ* = 1.27 and *M*_n_ of 3200 g/mol, which can be considered as an approximate value due to the differences in the hydrodynamic volumes of the calibration standards and the investigated copolymer. The ^1^H NMR spectrum in Figure 2 corroborates the structure of this copolymer as displayed in this Figure and Scheme 1. On the basis of the absence of the thiol (-SH) signal in the NMR spectrum at 1.58 ppm [48,55], it can be concluded that all the chain ends are terminated with acrylate groups of the PEGDA comonomer as well presented by the chemical shifts of the acrylate double bond protons at 5.84, 6.15 and 6.40 ppm. Thus, the number average molecular weight (*M*_n_) of the P(DODT-*co*-PEGDA) copolymer can be estimated from the corresponding integral values of the NMR signals compared to the integral values of the chain end olefinic protons. This led to *M*_n_ of 5400 g/mol for the prepared P(DODT-*co*-PEGDA) telechelic macromonomer.

For the synthesis of the conetworks, various compositions were applied in the range of 30–70 wt% P(DODT-*co*-PEGDA) macrocrosslinkers compared to the MA content in the feed as listed in Table 1. The concentration of the AIBN initiator was adjusted to a constant [M]/[I]^0.5^ ratio in order to obtain approximately the same average degree of polymerization of the monomer, which is sufficient to form a network in each case. Under these conditions, all the conetwork samples are formed with high gel fractions in the range of 66–83% (Table 1), confirming efficient crosslinking and successful conetwork syntheses. Decreasing gel fraction with increasing macrocrosslinker content is observed, which may be attributed to reduced mobility and diffusion limitations at higher copolymer concentrations. To estimate conetwork composition, the extracted materials were collected and analyzed by ^1^H NMR, as proposed by Petróczy et al. recently [25]. The conetwork composition was determined from the extracted material by ^1^H NMR spectroscopy. The weight fractions of PMA and P(DODT-*co*-PEGDA) in the extracted fractions were determined from the integral values of the corresponding NMR signals, and these were used to calculate the composition of the insoluble conetworks. These data, shown in Table 1, clearly indicate that PMA-*l*-P(DODT-*co*-PEGDA) conetworks with compositions close to the feed ratios are obtained.

### 2.2. Thermal Analysis of the PMA-l-P(DODT-co-PEGDA) Conetworks

The thermal behavior of the PMA-*l*-P(DODT-*co*-PEGDA) conetworks was examined by DSC and TGA to assess their phase transition and thermal stability, respectively. As shown in Figure 3 and Table 2, the P(DODT-*co*-PEGDA) copolymer exhibits glass transition temperature (*T*_g_) at −48 °C, while the other component, the PMA homopolymer has *T*_g_ at 15 °C. All the conetworks have one *T*_g_, which fall between the *T*_g_s of the components and depends on composition, i.e., the *T*_g_s decrease with increasing copolymer content, as shown in Figure 4. As also shown in this Figure, the physical blends of the components, i.e., the P(DODT-*co*-PEGDA) copolymer and the PMA homopolymer, possess two distinct *T*_g_s, one near to the *T*_g_ of PMA and another close to that of the copolymer, indicating minimal interfacial interaction, that is, immiscibility of these two polymers reflects macrophase separation due to the lack of covalent connectivity. In contrast, the single *T*_g_s of the conetworks indicate forced blending, i.e., miscibility of the components as a consequence of the covalent linkages in the conetworks. However, the *T*_g_s of the conetworks show negative deviations from both the linear additive rule and the Fox equation. This implies that attaching the P(DODT-*co*-PEGDA) segments to the PMA chains by covalent bonds introduces additional free volume and disrupts the self-association of the PMA chains, leading to a forced plasticizing effect. These observations demonstrate that covalent incorporation of the P(DODT-*co*-PEGDA) copolymers in the conetworks with PMA actively modifies segmental dynamics, allowing the thermal properties of the conetworks to be tuned by composition of such conetworks. In this context, it has to be mentioned that only one other similar case can be found in the literature for conetworks, i.e., for the poly(N-vinylimidazole)-*l*-poly(propylene glycol) conetworks, possessing forced blending [57].

The thermal stability and decomposition PMA-*l*-P(DODT-*co*-PEGDA) conetworks and their physical blends in comparison to that of the PMA homopolymer and P(DODT-*co*-PEGDA) copolymer were analyzed by thermogravimetric (TG) measurements. As shown in Figure 5 and Table 2, the thermal decomposition of all the samples begins around 300 °C and proceeds up to approximately 450 °C, following a two-step degradation pattern in the case of the blends and the conetworks as well. The initial, lower-temperature step is associated with the breakdown of the P(DODT-*co*-PEGDA) component, while the higher temperature step corresponds to the decomposition of the poly(methyl acrylate) fraction, as well evidenced by the coinciding maximal rate of decomposition with that of the corresponding components in the DTG curves (Figure 5b). This sequential degradation highlights the distinct thermal stability of each polymer segment in the conetworks. These observations suggest that the conetworks undergo a stepwise breakdown of the components by heating, which may have implications for their processing and potential high-temperature applications.

### 2.3. Swelling Behavior

Swelling experiments with hydrophilic solvents, such as water, methanol and ethanol, led to negligible swelling degrees in spite of the fact that both components, i.e., DODT and PEGDA, of the P(DODT-*co*-PEGDA) macrocrosslinker, contain two and three ethylene glycol (EG) units, respectively. This can be attributed to the disulfide and β-thioester linkages in addition to the low number of the moieties in the components of the copolymer. However, swelling in organic solvents, like THF, acetone and toluene, resulted in organogels with remarkable swelling degrees, as displayed in Figure 6. As shown in this Figure, the equilibrium swelling degree (*Q*) strongly depends on composition, which is closely related to the crosslinking density. As expected, *Q* decreases with increasing crosslinking density, i.e., increasing P(DODT-*co*-PEGDA) macrocrosslinker content in each investigated solvent. The data in Figure 6 also indicate that the equilibrium swelling degree can be regulated in these organogels by selecting the proper composition and swelling medium in a broad range by one order of magnitude. This means that *Q* is 0.6 in the case of the DA-70 sample (72 wt% copolymer content), while it is 8.7 in THF for the DA-30 conetwork sample (with 21 wt% copolymer).

The higher equilibrium swelling degree in THF can be attributed to the favorable solubility of both the macrocrosslinker and PMA in this solvent, on the one hand. On the other hand, the interaction of this solvent with the EG units in the P(DODT-*co*-PEGDA) macrocrosslinker promotes also stronger polymer–solvent interaction, resulting in enhanced swellability. In addition, THF also efficiently solvates disulfide and thioether groups as well. By contrast, the use of less polar toluene results in limited swelling and thus a more compact organogel.

### 2.4. Chemical Degradation of the Conetworks by Disulfide Reduction with Thiols and Hydrolysis of the β-Thioester Linkages by a Strong Base

Due to the presence of degradable units, that is, disulfide and β-thioester groups in the P(DODT-*co*-PEGDA) macrocrosslinker, the targeted degradability of the PMA-*l*-P(DODT-*co*-PEGDA) conetworks by treatment with excess thiol and sodium hydroxide solution was also explored. Treating the conetworks with thiols, like dithiothreitol (DTT) and 2-mercaptoethanol (2ME), in acetonitrile led to swelling and rapid disintegration of the conetwork gels into small swollen pieces. This indicates the break-up of the disulfide linkages in the P(DODT-*co*-PEGDA) macrocrosslinker in the conetworks by a disulfide–thiol redox exchange reaction. The incomplete disintegration of the conetworks is presumably due to the presence of some macrocrosslinkers without disulfide units, which may be the result of random distribution of disulfide units between the P(DODT-*co*-PEGDA) chains and/or to some extent of radical mediated disulfide fragmentation [58] during the conetwork formation by radical copolymerization. However, as the FTIR spectra in Figure 7 and Figure 8 proves, neither DTT nor 2ME have impact on the ester bonds in the conetworks, as the unchanged ester carbonyl stretching band at 1724 cm^−1^ upon treatment with these thiols clearly indicates. Adding NaOH solution to the conetwork treated with DTT and 2ME leads to complete break-up and dissolution of the gelled material. The FTIR spectra of the resulting product obtained after extraction with chloroform show the complete disappearance of the ester band, and the appearance of the asymmetric carboxylate stretch doublet at 1561 and 1643 cm^−1^, as displayed in Figure 7 and Figure 8, indicating complete hydrolysis of the ester groups in the conetwork. Comparing the IR spectra with that of the untreated conetwork, the treated samples exhibit also a broad signal at 3000–3600 cm^−1^, which is assigned to the stretching vibrations of -OH groups formed during the hydrolysis step.

Encouraged by the successful degradation of the PMA-*l*-P(DODT-*co*-PEGDA) conetworks after reacting with thiols followed by treatment with NaOH solution, adding only NaOH solution to the conetwork swollen in acetonitrile was also tested. This process led to complete disintegration of the conetwork. As shown in Figure 9, the carbonyl band of the ester group at 1724 cm^−1^ disappeared and the carboxylate bands together with the broad -OH band became present in the FTIR spectrum obtained after hydrolysis with NaOH. As displayed in Figure 10, the GPC curve of the chloroform-extracted material has a relatively large low-molecular-weight peak connected with two small higher-molecular-weight shoulders. Comparing the GPC curve of the product of the alkaline hydrolysis of the conetwork to that of the P(DODT-*co*-PEGDA) evidently indicates the successful hydrolysis and breaking of the macrocrosslinker by NaOH. The two smaller peaks in the GPC chromatogram of the degraded sample can be attributed to hydrolyzed chain segments with disulfide linkages.

## 3. Conclusions

The results presented in this study indicate that a novel, unique class of conetworks and their organogels with a broad range of composition were obtained by the radical copolymerization of methyl acrylate with sulfur-containing acrylate-telechelic P(DODT-*co*-PEGDA) macrocrosslinker. For this synthesis, first the scale-up of the preparation of the acrylate-telechelic P(DODT-*co*-PEGDA) macromonomer was successfully accomplished on the basis of a recent study by us [55] via copolymerization of DODT with PEGDA by simultaneous oxidative disulfide formation by DODT-DODT coupling, using H_2_O_2_ as oxidant and a thiol–ene Michael addition click reaction in the presence of PMDETA as a base catalyst for both reactions, that is, in a robust process (open air, room temperature) without any sophisticated preliminary synthetic steps. ^1^H NMR analysis proved the formation of the desired acrylate-telechelic P(DODT-*co*-PEGDA) copolymer with disulfide and β-thioester linkages. Using this copolymer as a macrocrosslinker successfully resulted in a series of PMA-*l*-P(DODT-*co*-PEGDA) conetworks with high gel fractions in a broad composition range of 20–70 wt% P(DODT-*co*-PEGDA) contents close to that of the feed ratios of the components.

Thermal analysis by DSC surprisingly resulted in one glass transition temperature for the conetworks composed of the otherwise immiscible PMA and P(DODT-*co*-PEGDA). This forced blending led to *T*_g_s with significant deviation from both the additive rule and the Fox equation for copolymers and miscible blends, indicating the remarkable effect of the chemical linking by the P(DODT-*co*-PEGDA) macrocrosslinker on the interaction and segment mobility of the polymer components in the conetworks. TGA measurements showed that both the components and the conetworks possess relatively high thermal stability up to ~300 °C, on the one hand. On the other hand, interestingly the DTG curves indicate that the P(DODT-*co*-PEGDA) and PMA components in the conetworks decompose separately at the same temperature range as the corresponding free constituents,; that is, the decomposition reactions of these two polymers do not interfere with each other.

Swelling studies demonstrated that the PMA-*l*-P(DODT-*co*-PEGDA) conetworks do not swell in polar protic solvents like water, methanol and ethanol, but swellable to high swelling degrees in organic solvents like THF, acetone and toluene. The swelling capacity of these novel organogels can be well controlled by composition, that is, by crosslinking density, in a broad range of ~60–900%.

Degradation of the PMA-*l*-P(DODT-*co*-PEGDA) conetworks was successfully achieved by treating the conetworks with thiols, such as dithiothreitol and 2-mercaptoethanol. However, these compounds led only to partial disintegration of the conetworks, presumably due to the relatively low disulfide content of the macrocrosslinker. Additional attempts of hydrolysis with NaOH led to complete disintegration of the conetworks, indicating the break-up of the β-thioester units in the macrocrosslinker as proved by FTIR measurements. Using only NaOH hydrolysis resulted in full disintegration of the conetworks via hydrolysis, leading to low-molecular-weight chloroform-extracted products.

As widely deemed, degradable polymers and polymer networks are viewed as one of the classes of high value-added and sustainable macromolecular materials (see e.g., Refs. [30,58,59,60,61,62,63,64,65,66,67,68,69,70,71,72,73,74,75,76,77,78] and references therein). The results of our study indicate that a broad range of degradable macromolecular materials can be obtained by applying the acrylate-telechelic P(DODT-*co*-PEGDA) copolymers as macrocrosslinkers or chain extenders, and thus to extend the family of degradable polymers and networks. Our findings also underline the distinctive features of the novel PMA-*l*-P(DODT-*co*-PEGDA) conetworks and their organogels, which enable to utilize these conetworks and the sulfur-rich P(DODT-*co*-PEGDA) and similar copolymers in a variety of advanced polymeric systems.

## 4. Materials and Methods

### 4.1. Materials

Methyl acrylate (MA) (≥99%), azobisisobutyronitrile (AIBN), poly(ethylene glycol) diacrylate (PEGDA) (*M*_n_ = 250 g/mol), tetrahydrofuran (THF), and 30 wt% aqueous hydrogen peroxide solution were obtained from Sigma Aldrich (Steinheim, Germany); 3,6-Dioxa-1,8-octane-dithiol (DODT) and *N*,*N*,*N*′,*N*″,*N*″-pentamethyldiethylenetriamine (PMDETA) were purchased from TCI (Zwijndrecht, Belgium). Tetrahydrofuran (THF) was distilled before use. PEGDA, methyl acrylate and DODT were purified by passing through basic alumina. AIBN was recrystallized from methanol before use. All other reagents were used as received.

### 4.2. Synthesis of the P(DODT-co-PEGDA) Copolymer

The copolymerization reaction between DODT and PEGDA for obtaining acrylate-telechelic poly(3,6-dioxa-1,8-octane-dithiol-*co*-poly(ethylene glycol) diacrylate) (P(DODT-*co*-PEGDA)) copolymer was carried out by a method reported recently by us [55]. Briefly, 14 mL PMDETA (0.067 mol) and 10 mL hydrogen peroxide aqueous solution (6.5 wt%) was added to a mixture of THF (200 mL), 16.6 mL DODT (0.1 mol) and 23.3 mL PEGDA (0.1 mol) at room temperature in open air. After 10 min of reaction time, the formed copolymer was precipitated in a large excess of methanol. Then, the solvent was decanted, and the copolymer was dried under vacuum at 40 °C until constant weight.

### 4.3. Synthesis of Poly(methyl acrylate)-l-P(DODT-co-PEGDA) Conetworks

For the synthesis of poly(methyl acrylate)-*l*-P(DODT-*co*-PEGDA) (PMA-*l*-P(DODT-*co*-PEGDA)) conetworks, predetermined amounts of methyl acrylate, P(DODT-*co*-PEGDA) copolymer and AIBN (from stock solution in THF), listed in Table 1, and additional THF to obtain 2 mL total volume were thoroughly mixed. The AIBN initiator was used in an amount to ensure a constant [monomer]/[initiator]^0.5^ concentration ratio in all cases. The resulting mixtures were heated in closed reaction vessels under nitrogen atmosphere in a glove box at 60 °C for 48 h. Subsequently, the solvent was allowed to evaporate in a hood for two days. This was followed by extraction with acetone and repeatedly exchanged for three days. Then, the conetworks were first air-dried, followed by drying in a vacuum oven by gradually decreasing the pressure until constant weight at room temperature.

### 4.4. Preparation of Blends of the P(DODT-co-PEGDA) Copolymer and Poly(methyl acrylate)

Physical blends of P(DODT-co-PEGDA) and PMA were prepared with P(DODT-*co*-PEGDA)/PMA weight ratios of 30/70 and 50/50. For the 30/70 blend, 0.06 g of P(DODT-co-PEGDA) and 0.14 g of PMA were used, while for the 50/50 blend, 0.10 g of P(DODT-co-PEGDA) and 0.10 g of PMA were measured and dissolved in 2 mL of THF, a common solvent for both components, to facilitate homogeneous mixing. The resulting mixtures were spread in glass Petri dishes and left for evaporation of THF under a fume hood. After solvent evaporation, the samples were further dried until constant weight in a vacuum oven at room temperature.

### 4.5. Swelling Measurements

The swelling behavior of the conetworks was evaluated by immersing dry polymer conetwork samples separately in THF, acetone, toluene and distilled water at room temperature. The swollen samples were removed several times and weighed until constant weights were achieved. In every case, swelling for ~2–3 h was sufficient to reach equilibrium swelling in the investigated solvents. The equilibrium swelling degree (*Q*) was determined according to the following equation:

*Q* = (*m*_s_ − *m*_d_)/*m*_d_ (1)

where *m*_s_ and *m*_d_ stand for the weights of the swollen and dry conetwork samples, respectively.

### 4.6. Degradation by Reduction of the Disulfide Units and Hydrolysis of the β-Thioester Linkages

The treatment of the conetwork samples with thiols was carried out similarly to that of the P(DODT-*co*-PEGDA) copolymer, as reported earlier by us [55]. In these cases, 0.1 g of conetwork samples was suspended in acetonitrile, and either DTT or 2-mercaptoethanol (2-ME) was added. The mixtures were stirred at room temperature for 48 h. Subsequently, 2 mL of 1 M aqueous NaOH solution was added under continuous nitrogen bubbling, and the mixtures were stirred to achieve hydrolytic cleavage at room temperature overnight. Then, chloroform was added, the phases were allowed to separate, and the organic phase was dried over anhydrous MgSO_4_. Finally, the solvent was removed under reduced pressure, and the residues were dried until constant weight. The disintegration of the conetwork was also attempted by using only hydrolysis with NaOH solution. In such reactions, 0.1 g conetwork was added to 5 mL acetonitrile followed by the addition of 3 mL NaOH solution of 1 M and allowed to react overnight. The sample work-up was the same as described above.

### 4.7. Characterizations

The nuclear magnetic resonance (^1^H NMR) spectra was obtained using a Varian 500 MHz spectrometer (Varian Inc., Palo Alto, CA, USA) in deuterated chloroform at 30 °C.

Gel permeation chromatography (GPC) was performed using a system equipped with a Waters 515 HPLC pump, a guard column, a series of Waters Styragel HR1, HR3, and HR4 columns, a Jetstream II Plus column thermostat (Waters, Milford, MA, USA), and an Agilent 1260 Infinity differential refractive index detector (Agilent Technologies, Santa Clara, CA, USA). THF was used as the eluent at a flow rate of 0.3 mL/min, and the measurements were carried out at 35 °C.

DSC measurements were conducted using a Mettler Toledo DSC821e instrument across a temperature range of −80 °C to 200 °C. A heating rate of 10 °C/min was used under nitrogen atmosphere. Glass transition temperatures (*T*_g_) were determined from the inflection points of the second heating curves.

Thermogravimetric analysis was performed on a TG-DSC system (Setaram LabsysEvo, Lyon, France). Samples were heated from 30 °C to 600 °C at a heating rate of 10 °C/min under a nitrogen flow of 90 mL/min.

The ATR FTIR spectra were recorded using a Bruker Tensor 27 FTIR spectrometer (Bruker Optics, Ettlingen, Germany) equipped with a Bruker Platinum ATR accessory. The dried samples were analyzed directly using the attenuated total reflectance (ATR) technique without further sample preparation. Each spectrum was collected by averaging 32 scans at a spectral resolution of 2 cm^−1^ over the wavenumber range of 4000–400 cm^−1^.

## Figures and Tables

**Figure 1:**
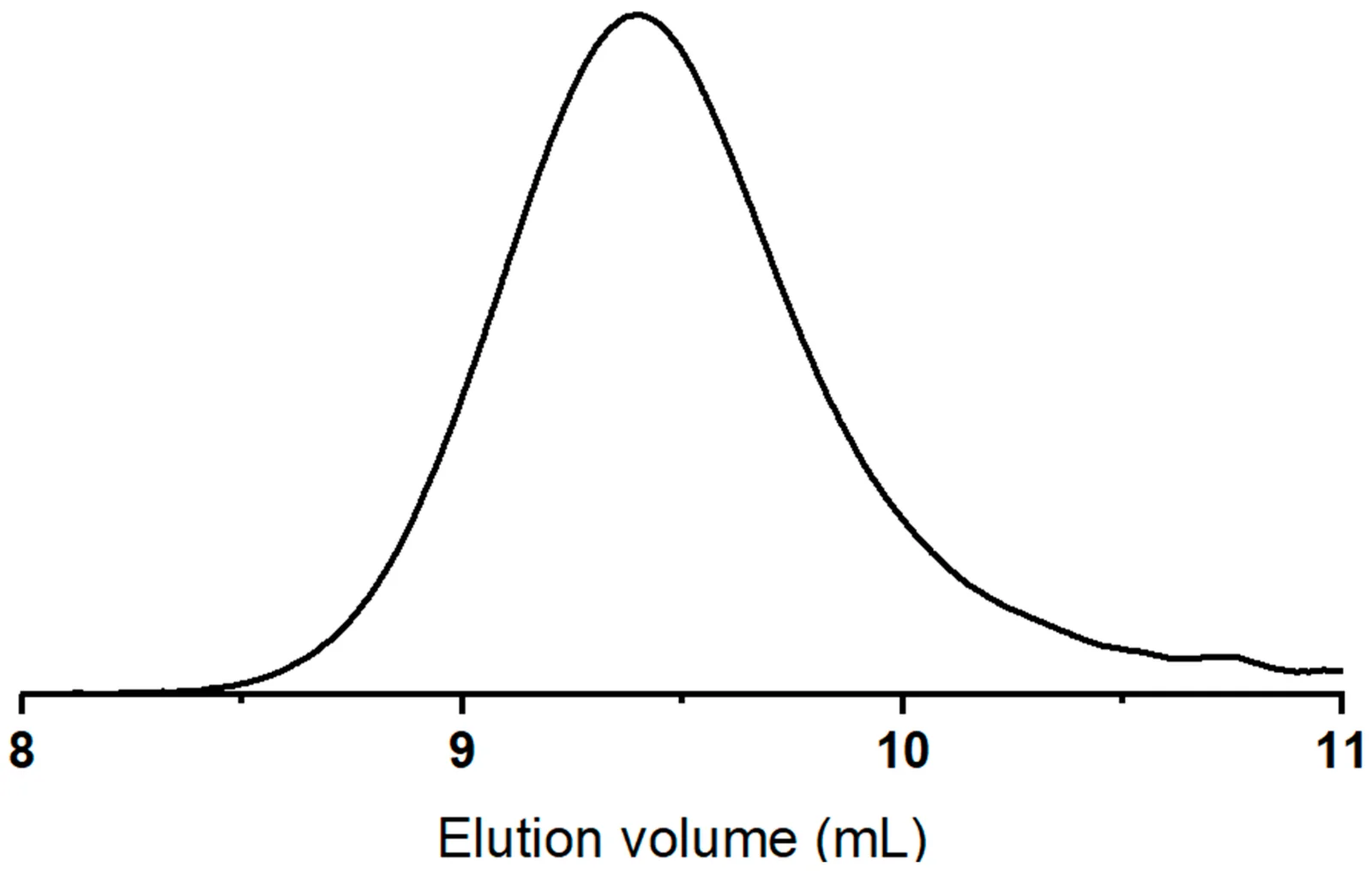
The GPC chromatogram of the acrylate-telechelic P(DODT-*co*-PEGDA) copolymer.

**Scheme 1:**
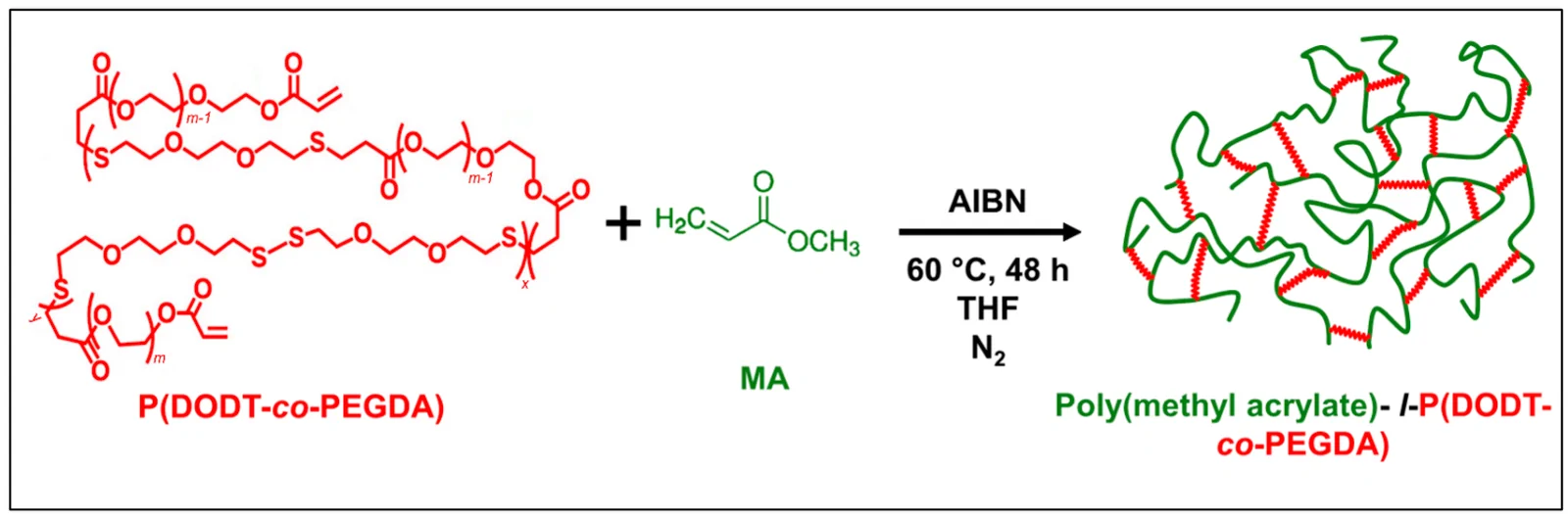
The synthesis route for the preparation of PMA-*l*-P(DODT-*co*-PEGDA) conetworks by radical copolymerization of methyl acrylate (MA) with the acrylate-telechelic P(DODT-*co*-PEGDA).

**Figure 2:**
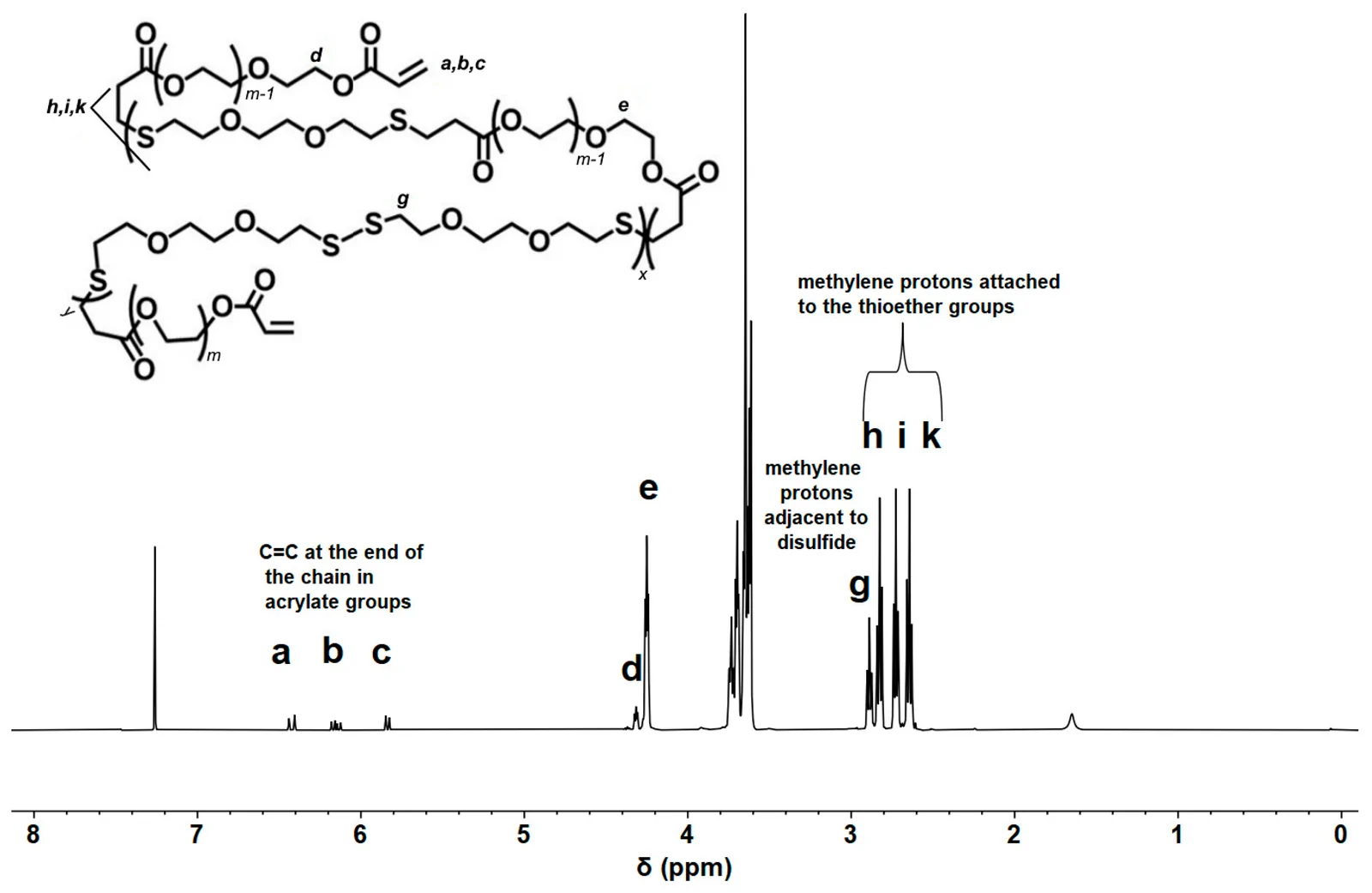
The ^1^H NMR spectrum of the acrylate-telechelic P(DODT-*co*-PEGDA) copolymer.

**Figure 3:**
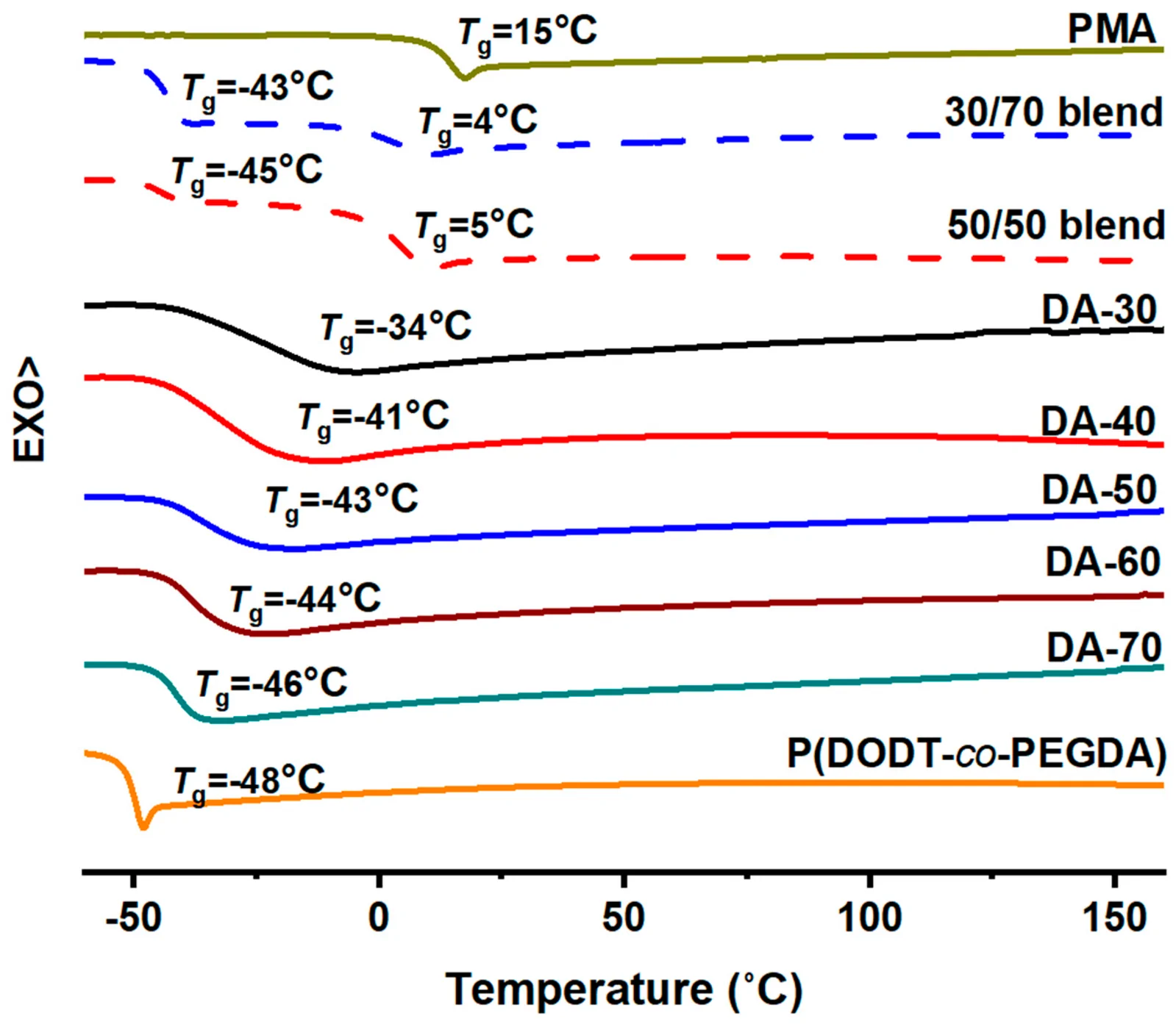
The DSC curves of the PMA-*l*-P(DODT-*co*-PEGDA) conetworks, the P(DODT-*co*-PEGDA)/PMA blends with 30 and 50 wt% P(DODT-*co*-PEGDA) content, the P(DODT-*co*-PEGDA) copolymer and the PMA homopolymer.

**Figure 4:**
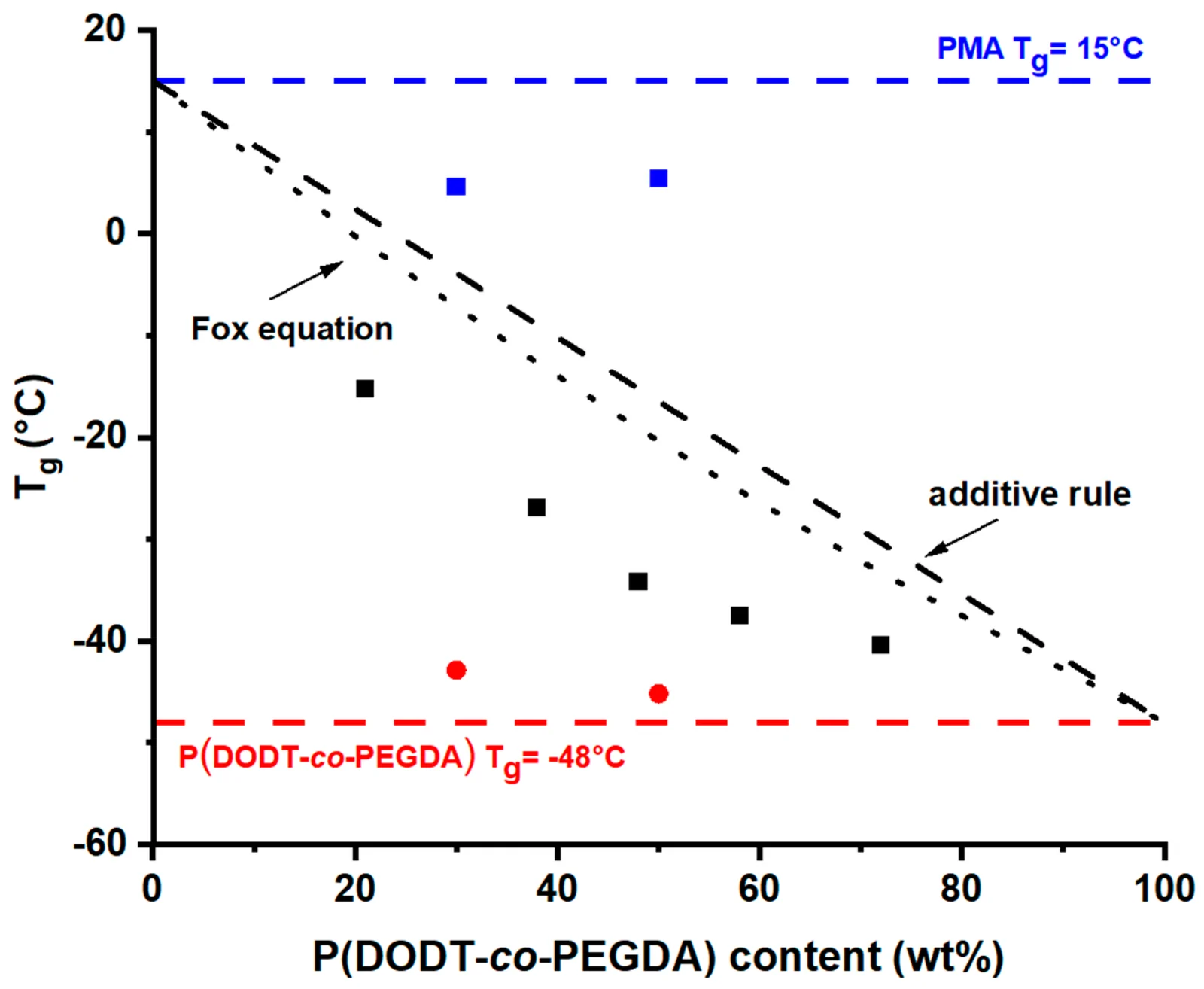
The glass transition temperatures (*T*_g_) of poly(methyl acrylate)-*l*-P(DODT-*co*-PEGDA) conetworks (black dots) as a function of the P(DODT-*co*-PEGDA) content. The dotted black curve represents the *T*_g_ values calculated using the Fox equation, and the dashed line indicates the *T*_g_ according to the linear additive rule of the components. The horizontal lines denote the *T*_g_ of the PMA (blue dashed line; 15 °C) and the P(DODT-*co*-PEGDA) (red dashed line; −48 °C). The red and blue dots represent the *T*_g_ values obtained for P(DODT-*co*-PEGDA) and PMA in physical blends.

**Figure 5:**
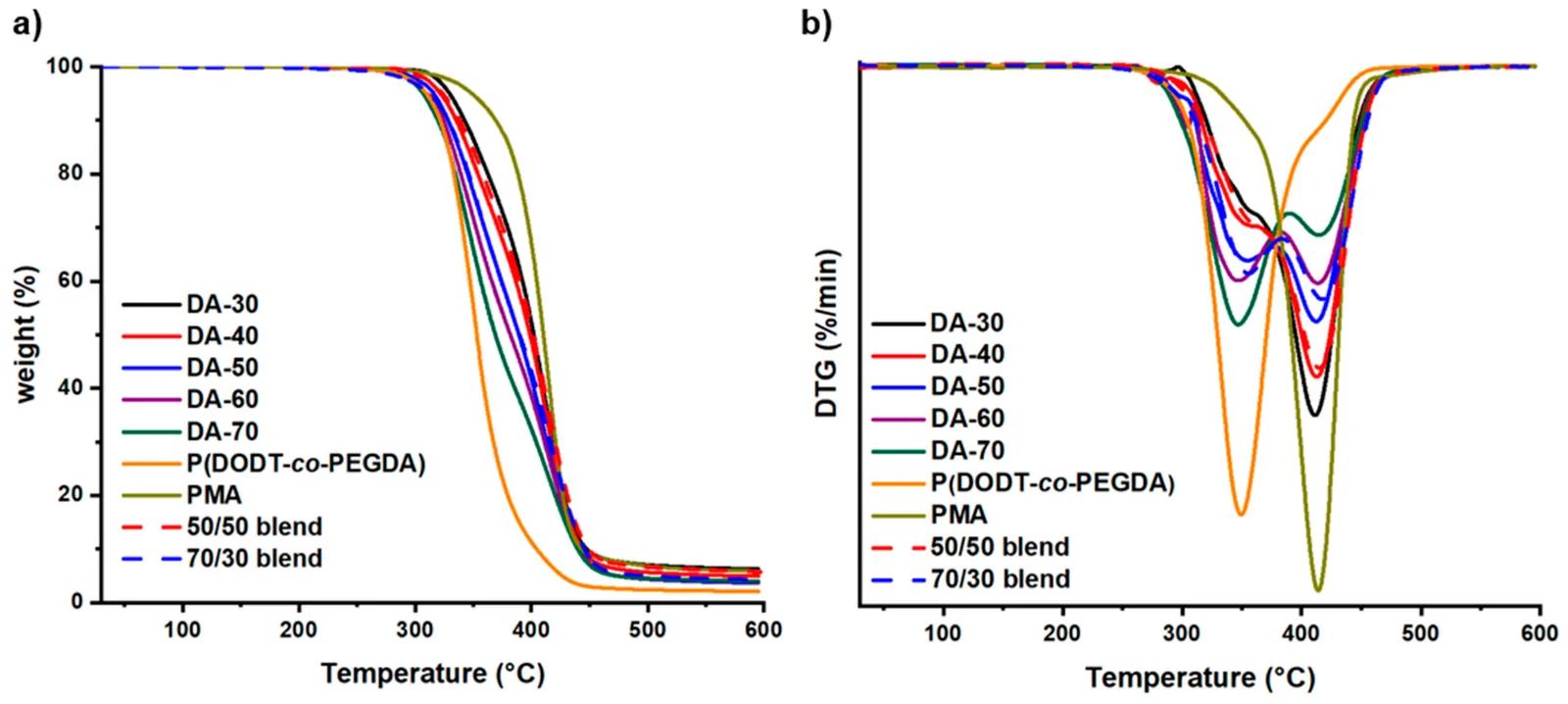
Thermal decomposition (**a**) and derivative thermogravimetric (DTG) curves (**b**) of the PMA-*l*-P(DODT-*co*-PEGDA) conetworks, the PMA homopolymer and the P(DODT-*co*-PEGDA) copolymer, and the PMA/P(DODT-*co*-PEGDA) blends with 30 and 50 wt% P(DODT-*co*-PEGDA) content (dotted lines) as a function of temperature (nitrogen atmosphere, 10 °C/min heating rate).

**Figure 6:**
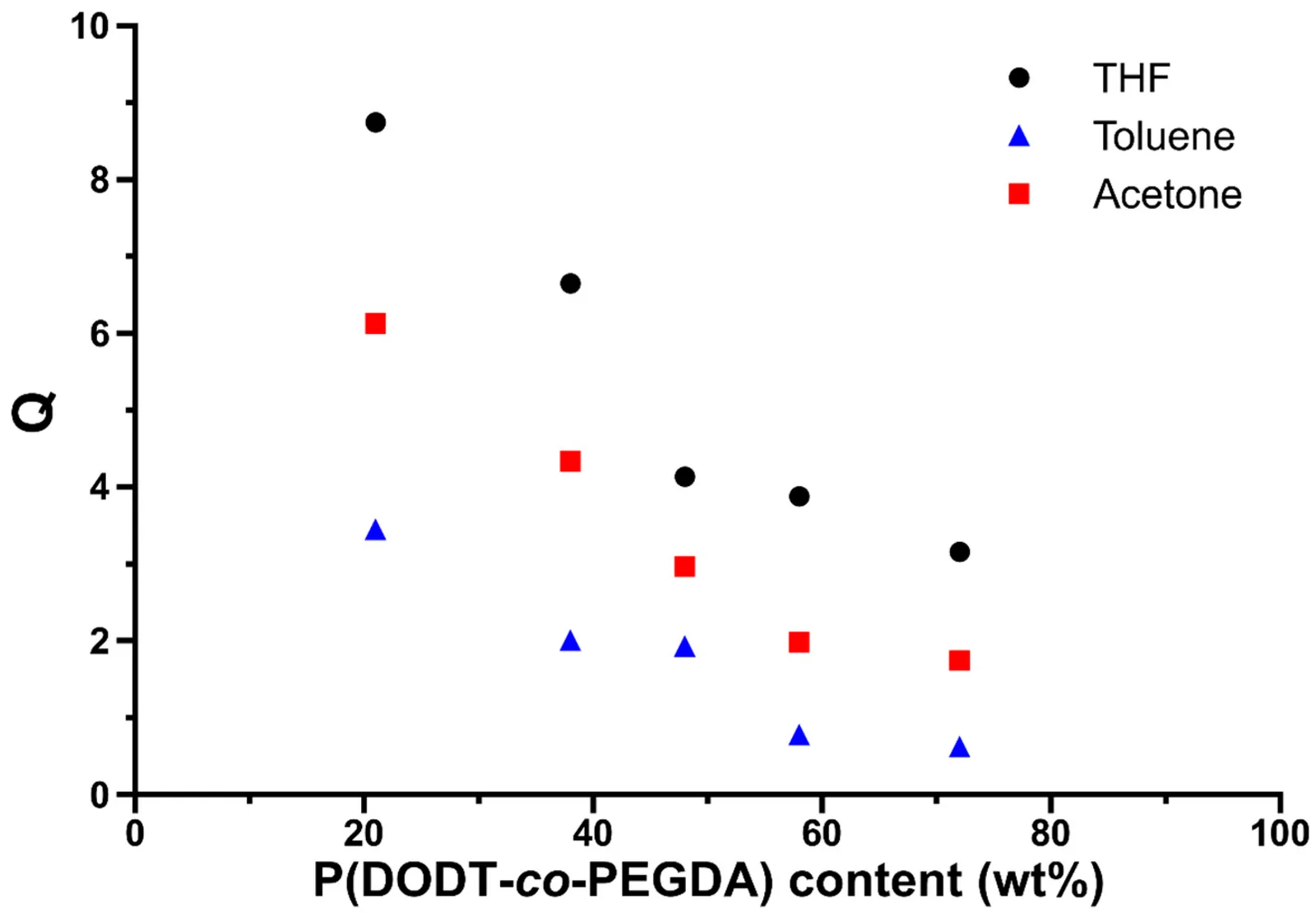
The equilibrium swelling degree (*Q*) of the PMA-*l*-P(DODT-*co*-PEGDA) conetworks as a function of the P(DODT-*co*-PEGDA) content in THF (closed black symbol), acetone (closed red symbols) and toluene (closed blue symbols).

**Figure 7:**
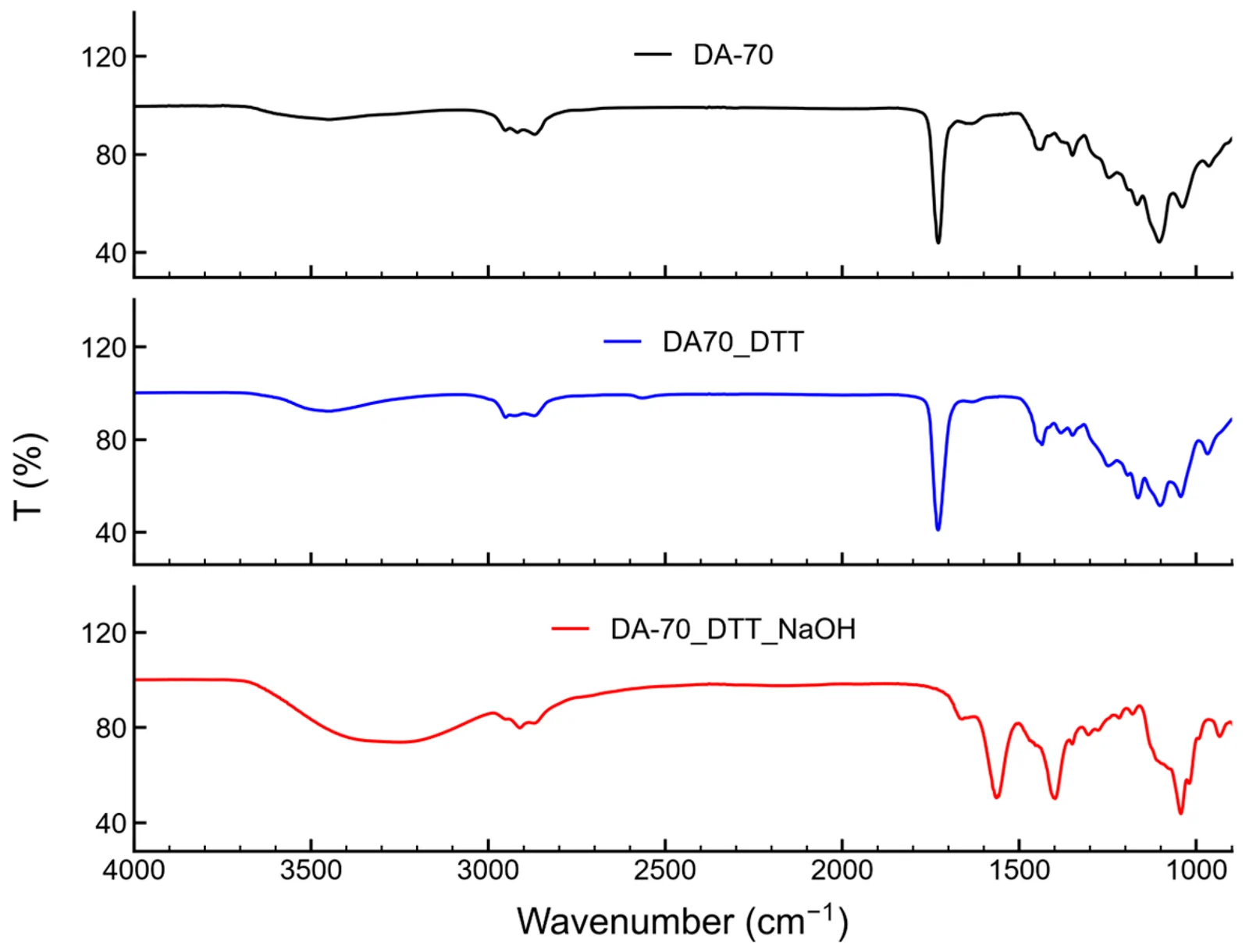
The FTIR spectra of the DA-70 PMA-*l*-P(DODT-co-PEGDA) conetwork sample (**top**), the conetwork treated with DTT (**middle**) and subsequently with NaOH solution (**bottom**).

**Figure 8:**
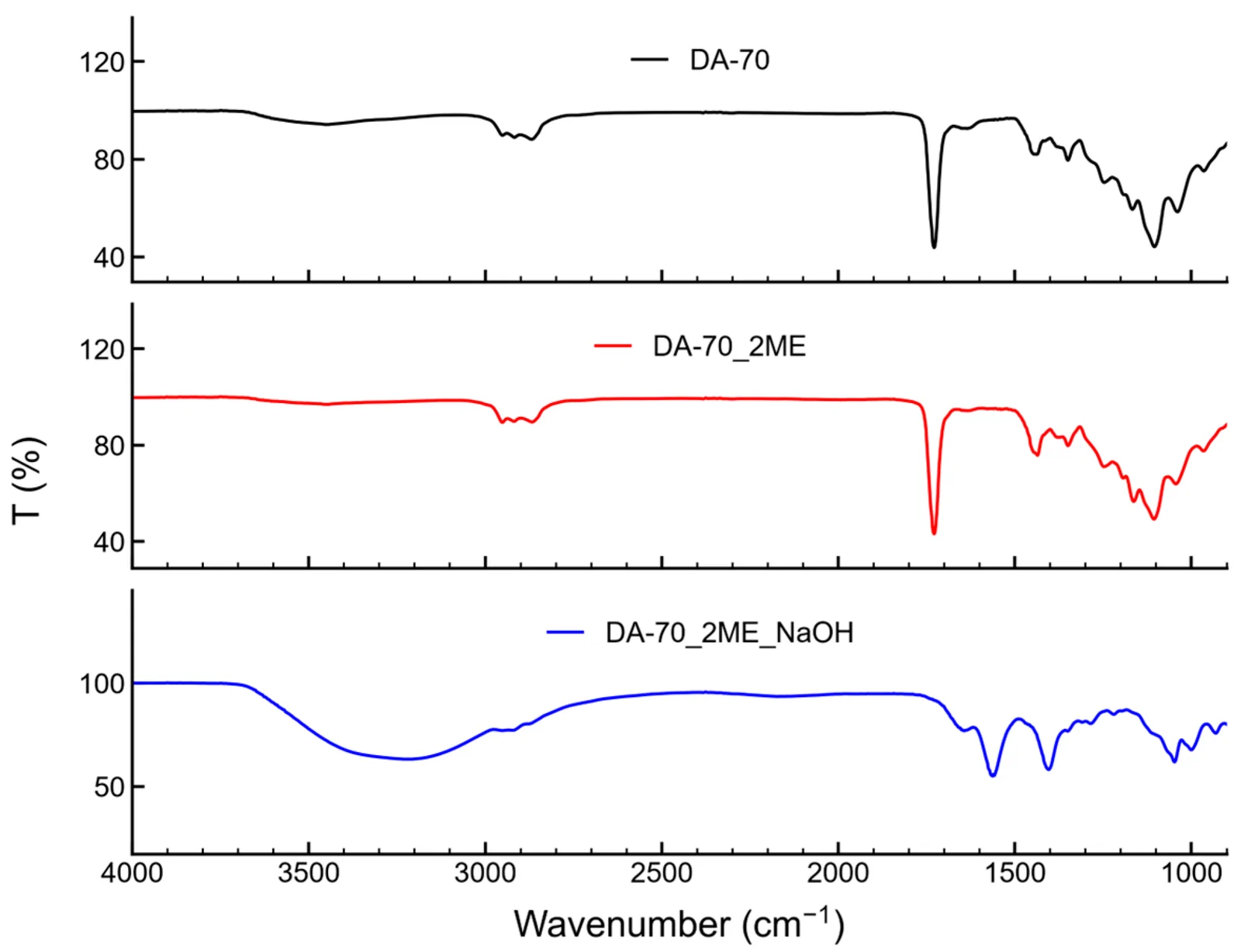
The FTIR spectra of the DA-70 PMA-*l*-P(DODT-co-PEGDA) conetwork sample (**top**), the conetwork treated with 2ME (**middle**) and subsequently with NaOH solution (**bottom**).

**Figure 9:**
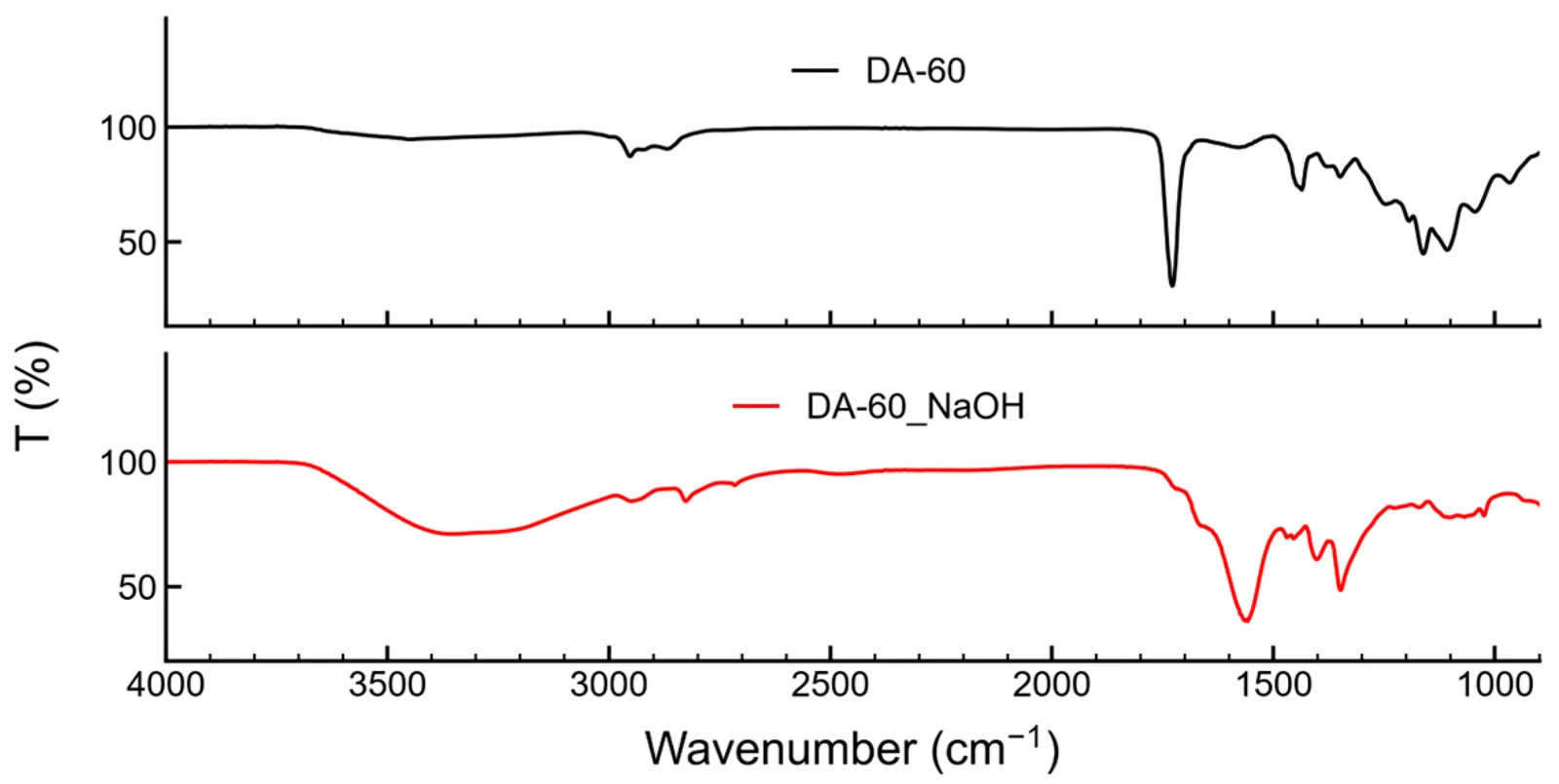
The FTIR spectra of the DA-60 PMA-*l*-P(DODT-*co*-PEGDA) conetwork sample before (**top**) and after treatment with NaOH solution (**bottom**).

**Figure 10:**
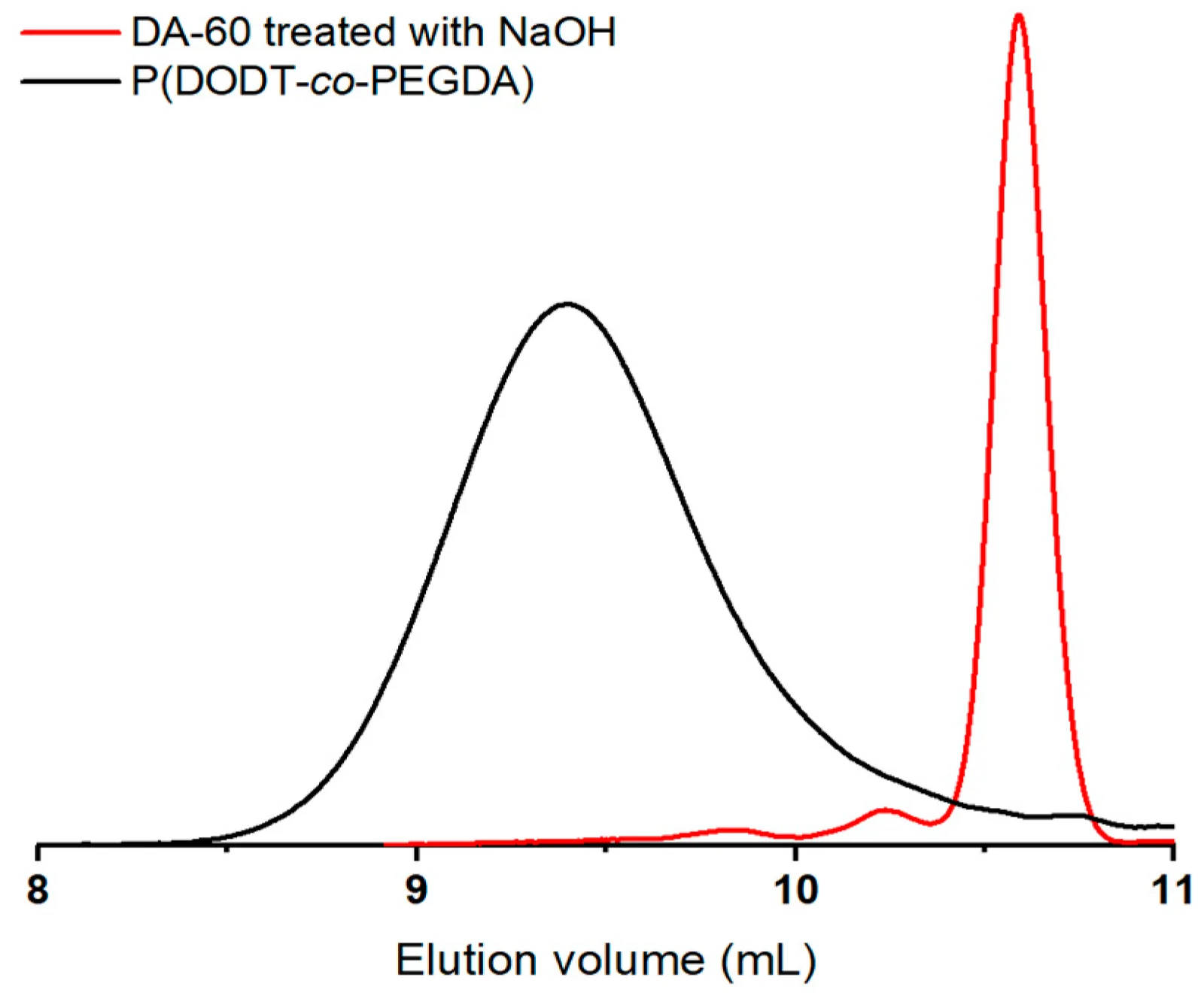
The GPC chromatogram of the P(DODT-*co*-PEGDA) macrocrosslinker and the product of the PMA-*l*-P(DODT-*co*-PEGDA) conetwork (sample DA-60) after treatment with NaOH solution.

**Table 1:** The applied amounts of the reactants in the synthesis of PMA-*l*-P(DODT-*co*-PEGDA) conetworks, and the gel fractions and copolymer contents in the resulting co-networks (THF solvent, total volume: 2 mL, 60 °C, 48 h).

Sample ID	Copolymer/MA Feed Ratio (wt%)	Copolymer (g)	MA (g)	AIBN (mg)	Gel Fraction (%)	Copolymer Content in Conetwork (wt%)
DA-30	30/70	0.30	0.70	18.98	83	21
DA-40	40/60	0.40	0.60	14.21	79	38
DA-50	50/50	0.50	0.50	10.07	75	52
DA-60	60/40	0.60	0.40	6.66	68	58
DA-70	70/30	0.70	0.30	3.94	66	72

**Table 2:** The glass transition temperatures (*T*_g_) and the characteristic thermal decomposition temperatures (*T*_5_, *T*_10_ and *T*_max_) of the PMA-*l*-P(DODT-*co*-PEGDA) conetworks, the P(DODT-*co*-PEGDA)/PMA blends, the P(DODT-*co*-PEGDA) copolymer and the PMA homopolymer (*T*_5_, *T*_10_ and *T*_max_ stand for the temperatures at 5%, 10% weight loss and the maximal rate of decomposition, respectively).

Sample ID	Copolymer Content in Conetwork (wt%)	*T*_g_ (°C)	*T*_5_ (°C)	*T*_10_ (°C)	*T*_max_ (°C)
DA-30	21	−34	314.0	331.5	353.6, 413.0
DA-40	38	−41	318.7	333.8	345.1, 414.1
DA-50	52	−43	314.8	327.6	345.7, 415.2
DA-60	58	−44	310.0	324.0	349.2, 415.8
DA-70	72	−46	303.1	317.7	348.9, 418.6
Blend-30/70 ^a^	30	4, −43	311.6	328.5	355.6, 417.2
Blend-50/50 ^a^	50	5, −45	323.8	338.6	364.0, 414.8
Copolymer ^b^	100	−48	309.8	321.6	349.5
PMA	-	15	348.8	369.4	413.9

^a^ P(DODT-*co*-PEGDA)/PMA physical blend. ^b^ P(DODT-*co*-PEGDA) copolymer.

## Data Availability

The original contributions presented in this study are included in the article. Further inquiries can be directed to the corresponding author.
